# How amenable is type 2 diabetes treatment for precision diabetology? A meta-regression of glycaemic control data from 174 randomised trials

**DOI:** 10.1007/s00125-023-05951-2

**Published:** 2023-06-20

**Authors:** Oliver Kuss, Marie Elisabeth Opitz, Lea Verena Brandstetter, Sabrina Schlesinger, Michael Roden, Annika Hoyer

**Affiliations:** 1 Institute for Biometrics and Epidemiology, German Diabetes Center, Leibniz Institute for Diabetes Research at Heinrich Heine University Düsseldorf, Düsseldorf, Germany; 2 Centre for Health and Society, Faculty of Medicine, Heinrich Heine University Düsseldorf, Düsseldorf, Germany; 3 German Center for Diabetes Research, Partner Düsseldorf, München-Neuherberg, Germany; 4 Department of Statistics, Ludwig-Maximilians-University Munich, Munich, Germany; 5 Institute for Clinical Diabetology, German Diabetes Center, Leibniz Center for Diabetes Research at Heinrich Heine University Düsseldorf, Düsseldorf, Germany; 6 Department of Endocrinology and Diabetology, Medical Faculty and University Hospital Düsseldorf, Heinrich Heine University Düsseldorf, Düsseldorf, Germany; 7 Biostatistics and Medical Biometry, Medical School EWL, Bielefeld University, Bielefeld, Germany

**Keywords:** Dipeptidyl peptidase-4 inhibitors, Glucagon-like peptide 1, HbA_1c_, Meta-regression, Precision medicine, Sodium–glucose transporter 2 inhibitors, Type 2 diabetes mellitus

## Abstract

**Aims/hypothesis:**

There are two prerequisites for the precision medicine approach to be beneficial for treated individuals. First, there must be treatment heterogeneity; second, in the case of treatment heterogeneity, we need to detect clinical predictors to identify people who would benefit from one treatment more than from others. There is an established meta-regression approach to assess these two prerequisites that relies on measuring the variability of a clinical outcome after treatment in placebo-controlled randomised trials. Our aim was to apply this approach to the treatment of type 2 diabetes.

**Methods:**

We performed a meta-regression analysis using information from 174 placebo-controlled randomised trials with 178 placebo and 272 verum (i.e. active treatment) arms including 86,940 participants with respect to the variability of glycaemic control as assessed by HbA_1c_ after treatment and its potential predictors.

**Results:**

The adjusted difference in log(SD) values between the verum and placebo arms was 0.037 (95% CI: 0.004, 0.069). That is, we found a small increase in the variability of HbA_1c_ values after treatment in the verum arms. In addition, one potentially relevant predictor for explaining this increase, drug class, was observed, and GLP-1 receptor agonists yielded the largest differences in log(SD) values.

**Conclusions/interpretation:**

The potential of the precision medicine approach in the treatment of type 2 diabetes is modest at best, at least with regard to an improvement in glycaemic control. Our finding of a larger variability after treatment with GLP-1 receptor agonists in individuals with poor glycaemic control should be replicated and/or validated with other clinical outcomes and with different study designs.

**Funding:**

The research reported here received no specific grant from any funding agency in the public, commercial or not-for-profit sectors.

**Data availability:**

Two datasets (one for the log[SD] and one for the baseline-corrected log[SD]) to reproduce the analyses from this paper are available on https://zenodo.org/record/7956635.

**Graphical Abstract:**

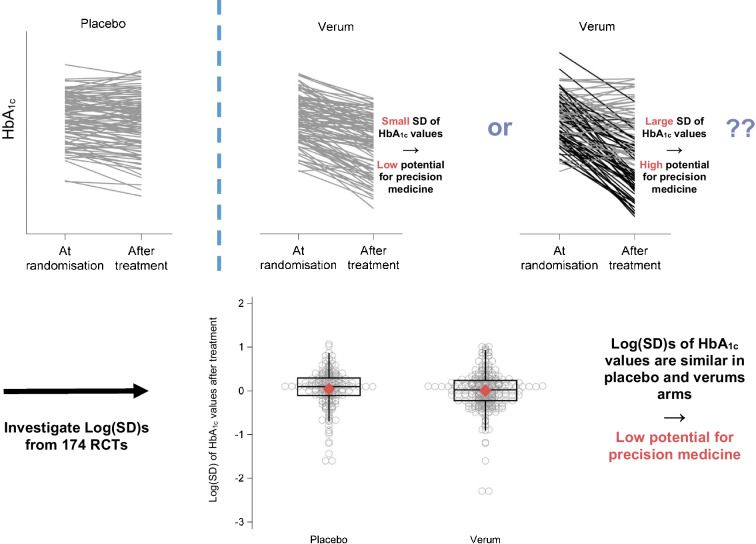

**Supplementary Information:**

The online version of this article (10.1007/s00125-023-05951-2) contains peer-reviewed but unedited supplementary material.


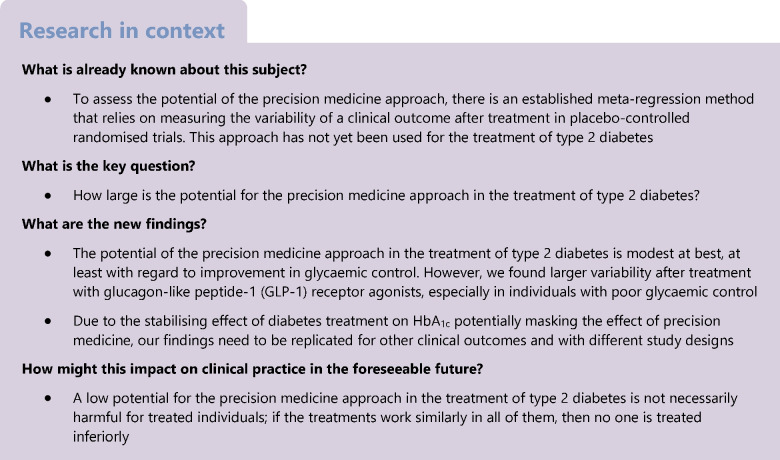


## Introduction

In 1999, F. S. Collins, the then leader of the Human Genome Project, announced a genetic revolution in medicine, fundamentally changing the diagnosis, treatment and prevention of multiple diseases [1]. With respect to treatment, the resulting ‘individualised medicine’ would use identified human genetic variation to subclassify diseases and tailor therapies to the individual patient because there ‘may be large differences in the effectiveness of medicines from one person to the next’ [1].

Tremendous amounts of knowledge with respect to genetics and other biomarkers have been assembled since then, more recently also in diabetology. Indeed, and as emphasised by J. M. Dennis [2], the treatment of type 2 diabetes seems to be particularly well-suited for a precision medicine approach. After initial treatment with metformin, a number of drugs with different mechanisms of action are available and there is no clear ‘best’ overall treatment, except for in the treatment of a small group of individuals with specific complications. In addition, there is large heterogeneity in the clinical phenotype of type 2 diabetes [3], making it plausible that people with different underlying pathophysiologies will have varying responses to different drugs. Indeed, the idea of precision treatment has a strong momentum in diabetes research. Two leading diabetes societies, the ADA and the EASD, founded a ‘Precision Medicine in Diabetes Initiative’, issued a common consensus report [4] and recently reported the progress of the initiative and its future vision [5]. The official journal of the EASD, *Diabetologia*, published a special issue on precision medicine [6], collecting 16 reviews written by well-recognised experts on various aspects of precision medicine in diabetes. With a view towards future precision-based pharmacological treatment of type 2 diabetes, Florez and Pearson [7] introduced a roadmap to precision medicine becoming the standard of care and determined what additional work is needed.

A prerequisite for precision medicine is that there is treatment heterogeneity, i.e. that a treated person responds differently to different treatments. From a methodological point of view, this assumption proclaims an interaction between treatment and person. However, it is largely unknown that this interaction cannot be observed directly from a standard RCT, where only an average treatment effect can be observed. Instead, repeated-crossover or *N*-of-1 trials are needed [8, 9], in which individuals are treated at least twice with at least one of the treatments under study. Moreover, differences in outcomes between treated individuals are not necessarily caused by a heterogeneous treatment effect but may also arise from random variation within and/or differences between treated individuals [10].

It is also often overlooked that another prerequisite is necessary for making precision medicine clinically useful [11]. Even if there is real treatment heterogeneity, predictors (e.g. age, sex, HbA_1c_) must be available to identify people who would benefit more from a given treatment than from others.

**Fig. 1:**
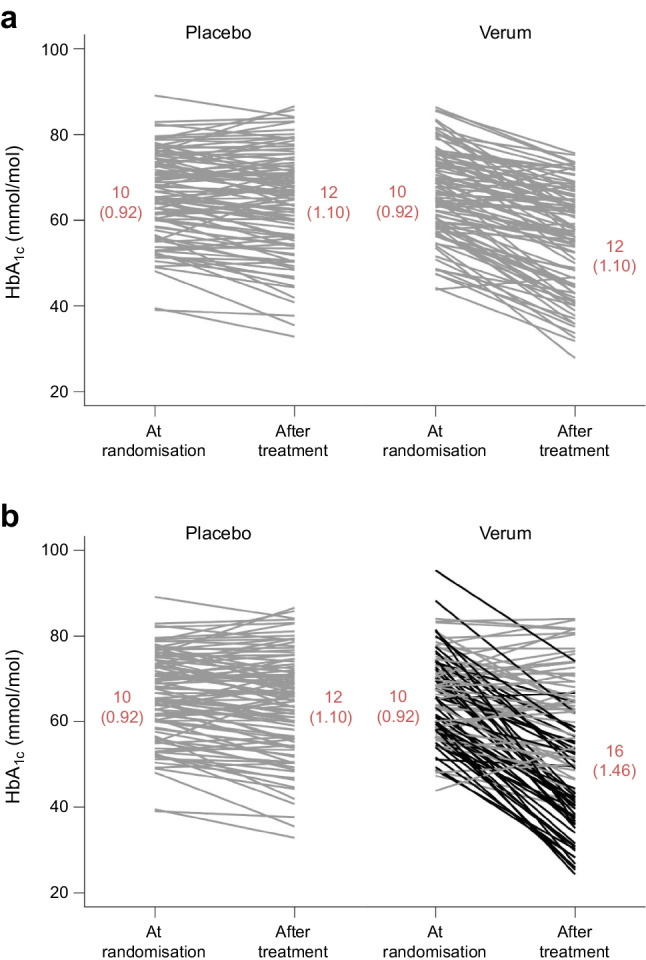
Results of two fictitious but realistic randomised trials that compare a placebo to a verum treatment. Given are 200 individual HbA_1c_ trajectories from the baseline to the HbA_1c_ value after treatment and (in red) the corresponding standard deviations of HbA_1c_ values at the two different time points and treatment arms. In both trials, and as a consequence of randomisation, the SD of HbA_1c_ values at baseline is identical (10 mmol/mol [0.92%]). (**a**) In this trial there was no differential heterogeneity between treatments, and the SD of HbA_1c_ values after treatment equals 12 mmol/mol (1.10%) in both treatment arms. Please note that there is a treatment effect, and verum treatment leads to a stronger reduction in HbA_1c_ values. However, this is no contradiction to treatment heterogeneity being absent. Treatment heterogeneity is measured by the interaction between treatment and person and not by the treatment itself. (**b**) In this trial there was heterogeneity between treated individuals. Indeed, there is a group of non-responders (grey lines) and a group of ‘super responders’ (black lines) in which the HbA_1c_ values are lowered considerably more strongly. As a consequence, the SD in the verum group is considerably larger (16 mmol/mol [1.46%])

We are not aware of any previous work that attempted to assess the potential of precision medicine in diabetology by checking these two prerequisites of treatment heterogeneity and available predictors. Recently, such methods have been proposed and used in other disciplines, e.g. in psychiatry [10, 12–15] and pain research [16]. These methods rely on the basic idea that if there is real treatment heterogeneity, the variability of outcomes after treatment in a randomised, placebo-controlled trial will be larger in the verum (i.e. active treatment) arms than in the placebo arms (see Fig. 1 for a detailed explanation). In other words, if we observe larger variability in outcome values after treatment in the verum arm than in the placebo arm, there is treatment heterogeneity and thus a greater potential for a precision medicine approach. To investigate the second prerequisite of the precision medicine approach, the availability of predictors for treatment heterogeneity, we can additionally explore interactions of predictors and treatment.

To assess whether treatment of type 2 diabetes is amenable to the precision medicine approach, we report here on a meta-regression analysis of randomised, placebo-controlled trials aimed at treatment of type 2 diabetes with respect to the variability of HbA_1c_ values after treatment and its potential predictors.

## Methods

### Included trials

Our study population comprised all RCTs from three recent systematic reviews [17–19] that compared treatments for type 2 diabetes (alpha-glucosidase inhibitors, dipeptidyl peptidase-4 (DPP-4) inhibitors, glucagon-like peptide-1 (GLP-1) receptor agonists, metformin, sodium–glucose cotransporter-2 (SGLT-2) inhibitors, sulfonylureas, thiazolidinediones, combination therapies or others) to placebo and reported on the outcome of glycaemic control as assessed by HbA_1c_ (in %). Multiple treatment arms from the same trial were allowed where they could result from different drugs and/or different doses of the same drug and/or different application forms being compared. Trials were also eligible if placebo and verum were given as a randomised add-on to a pre-existing diabetes treatment.

### Outcomes

Our primary outcome was the variability of HbA_1c_ values, measured as the logarithm of the standard deviation (log[SD]), after treatment in each trial arm. As these log(SD) values were not always reported in the original trial publications, we used elementary conversion formulas from standard errors to SDs and from 95% confidence intervals for the mean HbA_1c_ value to arrive at SDs. In addition, when only medians and/or quartiles and/or minima/maxima of HbA_1c_ values were given in the original trial publications, we used the formulas given by Luo et al [20] and McGrath et al [21] to achieve means and SDs of HbA_1c_ values. We extracted values from the text as well as from figures in the original trial publications. Only unadjusted log(SD) values were extracted, and we did not include information from analyses using ANCOVA techniques. If HbA_1c_ values were reported on the mmol/mol scale, we used the formula HbA_1c_(%)=HbA_1c_(mmol/mol) × 0.0915 + 2.15 to achieve HbA_1c_ values on the per cent scale.

Two types of log(SD) values after treatment were reported in the original trial publications. The first type of log(SD) values used the originally observed HbA_1c_ values after treatment, and the second type used baseline-corrected HbA_1c_ values. Baseline correction here means that the baseline HbA_1c_ value (i.e. *before* treatment) for each individual participant was subtracted from its HbA_1c_ value *after* treatment, and only then were the log(SD) values of the resulting baseline-corrected HbA_1c_ values after treatment computed and reported. Both types of log(SD) values, raw and baseline-corrected, are of interest, and they are essentially comparable on the same scale; trials reported on one or on both of them. For the sake of brevity, we focus on the raw log(SD) in the main paper and give the results for the baseline-corrected log(SD) in the electronic supplementary material (ESM).

### Data extraction

In terms of data extraction, data from the first Palmer et al review [17] were already available from a previous project [22] and had been extracted by two independent reviewers. All remaining trial publications were read by one of two reviewers (MEO or LVB), and each of them validated a small sample of publications of the other reviewer. Both reviewers were in regular contact with OK and AH to calibrate and harmonise data extraction.

### Statistical analysis

To assess the first prerequisite (treatment heterogeneity, defined as larger variability of HbA_1c_ values after treatment in verum arms compared with placebo arms) we used the ‘arm-based’ model of Nakagawa et al [23], where each trial arm is considered a single observation. To be concrete, we fitted a weighted meta-regression model with the bias-corrected (23, equation 7) outcome ${\mathrm{log(}\mathrm{SD)}}_{{\mathrm{HbA}}{\mathrm{1c}}}\mathrm{+}{1}/\mathrm{(2}{{n}}\mathrm{-1)}$ as the response variable, where ${\mathrm{n}}$ denotes the sample size in the respective trial arm, and ${1}/\mathrm{(2}{{n}}\mathrm{-1)}$ is the bias correction. Fixed effect covariates in this meta-regression model were: (1) the treatment (verum vs placebo); and (2) the logarithm of the mean HbA_1c_ value (${\mathrm{log(}\overline{\mathrm{x}}\mathrm{)}}_{\mathrm{HbA}\mathrm{1c}}$) after treatment in the respective trial arm. To account for correlations between treatment arms from the same trial, we included a random intercept for the trial in the meta-regression model. Finally, with the aim of adjusting for the different sample sizes of trial arms, we followed the standard inverse-variance principle in meta-analysis and weighted each observation by $\mathrm{(2}{{n}}\mathrm{-1)}$, the inverse variance of the bias-corrected estimate of log(SD) (23, equation 8).

The key parameter of interest in this meta-regression model is the regression coefficient for the treatment effect. If this value is 0, then there is no difference between the verum and placebo arms with respect to their log(SD) values, although there was proper adjustment for the size of the treatment effect (via the log(mean)), the correlations within trials (via the random intercept) and the different sample sizes in trial arms (via inverse-variance weighting). Values above 0 indicate a larger variability in the verum arms, i.e. treatment heterogeneity, and thus a potential for precision treatment in individuals with type 2 diabetes.

To assess the second prerequisite (identification of predictors to explain treatment heterogeneity), we used a separate meta-regression model for each individual predictor. To this end, the meta-regression model as described in the previous paragraph was extended by an additional interaction term of the respective predictor with treatment. Evaluated predictors were mean age, proportion of male participants, mean BMI, mean known disease duration and the mean HbA_1c_ (in %) of populations at baseline in the respective trial arm. We further assessed drug class, the duration of the trial and the year in which the trial was performed as potential predictors.

SAS, Version 9.4 (SAS Institute, Cary, NC, USA), was used for data management and analysis. As the study does not include personalised data, we did not seek a vote from an ethics committee. The study was not preregistered and had no previously published protocol.

## Results

After removing duplicate trials from the three systematic reviews, 382 RCTs with at least one placebo arm were eligible. Four trials had to be excluded because the full texts could not be retrieved and one trial had to be excluded because it lacked information on the placebo arm. Of the remaining 377 trials, 193 did not report on the outcome log(SD), 30 did not report on the sample size and 14 did not report on the mean HbA_1c_ value after treatment. A trial could have no information on more than one of the three items (no log(SD), no sample size, or no mean HbA_1c_ value), so we ended up with a final dataset of 450 trial arms (272 verum arms with 52,195 participants and 178 placebo arms with 34,745 participants) from 174 different trials.

**Fig. 2:**
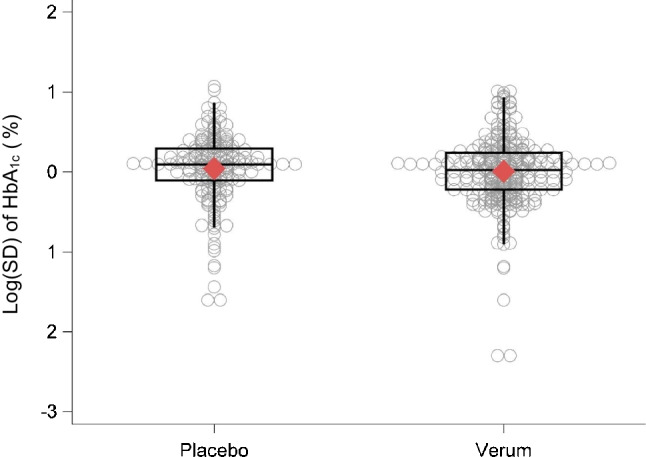
Boxplots and observed values for the log(SD) of HbA_1c_ values after treatment, separately for the verum and placebo arms. Bottom and top edges of the boxes show the first (Q1) and third (Q3) quartile, while the horizontal line inside the box indicates the median value. The red diamond within the boxes shows the respective mean value. The whiskers that extend from a box indicate the range of values that are outside of the intra-quartile range. Note that these boxplots do not adjust for the mean HbA_1c_, the sample size or for the correlation within trials

The description of trial populations is given in Table 1. At baseline and as a consequence of randomisation, populations in the placebo and verum arms were similar with respect to mean age, proportion of male participants, mean BMI and mean known disease duration. The most frequently used treatments were DPP-4 inhibitors (in 58 verum arms), GLP-1 receptor agonists (56) and SGLT-2 inhibitors (42). The median mean HbA_1c_ values were 66.1 mmol/mol (8.2%) before treatment, and 63.9/56.4 mmol/mol (8.0%/7.3%) in the placebo/verum arms after treatment, indicating a clear overall beneficial effect of verum treatments. In terms of the primary outcome, the median log(SD) of HbA_1c_ values after treatment was 0.10%/0.02% in the placebo/verum arms pointing to larger variability of HbA_1c_ in the placebo arms. Regarding the complete distributions of log(SD) values, no differences were observed between the verum and placebo arms (Fig. 2). However, these boxplots are not adjusted for the mean HbA_1c_, the sample size or the correlation within trials.

**Table 1:** Description of included trial arms, separated by placebo and verum arms

	Placebo (*N*=178 arms)		Verum (*N*=272 arms)	
Variable	Number of missing arms	Median (Min/Q1/Q3/Max)	Number of missing arms	Median (Min/Q1/Q3/Max) or Number (%)
Mean age at baseline (in years)	7	57.0 (39.5/55.0/59.0/74.4)	11	56.5 (38.8/54.8/58.9/74.0)
Proportion of male participants at baseline (in %)	9	54.3 (0.5/48.9/61.1/100)	16	55.3 (0.4/48.9/61.1/100)
Mean BMI at baseline (in kg/m^2^)	6	30.6 (22.9/27.6/32.3/41.6)	10	30.9 (23.5/28.3/32.3/40.7)
Mean known disease duration at baseline (in years)	35	8.0 (0.0/5.6/10.4/19.6)	57	7.3 (0.0/5.0/9.8/16.4)
Mean HbA_1c_ at baseline (in mmol/mol)	0	66.1 (38.8/62.8/71.6/112.0)	0	66.1 (43.5/62.8/70.6/116.4)
Mean HbA_1c_ at baseline (in %)	0	8.2 (5.7/7.9/8.7/12.4)	0	8.2 (6.1/7.9/8.6/12.8)
Year	0	2013 (1987/2006/2016/2020)	0	2012 (1987/2006/2016/2020)
Treatment (drug class)				
Alpha-glucosidase inhibitors	--	--	0	22 (8)
DPP-4 inhibitors	--	--	0	58 (21)
GLP-1 receptor agonists	--	--	0	56 (21)
Metformin	--	--	0	22 (8)
SGLT-2 inhibitors	--	--	0	42 (15)
Sulfonylureas	--	--	0	8 (3)
Thiazolidinediones	--	--	0	47 (17)
Combination therapies	--	--	0	10 (4)
Others	--	--	0	7 (3)
Duration of treatment (in weeks)	0	26.0 (16/24/36/260)	0	26.0 (16/24/30/260)
Number of treated individuals	0	83 (5/41/137/7998)	0	108 (6/53.5/164.5/8078)
Mean HbA_1c_ after treatment (in mmol/mol)	0	63.9 (36.6/59.8/70.2/134.0)	0	56.4 (38.8/52.4/61.2/117.8)
Mean HbA_1c_ after treatment (in %)	0	8.0 (5.5/7.6/8.6/14.4)	0	7.3 (5.7/6.9/7.7/12.9)
Log(mean) of HbA_1c_ after treatment (in mmol/mol)	0	4.2 (3.6/4.1/4.3/4.9)	0	4.0 (3.7/4.0/4.1/4.8)
Log(mean) of HbA_1c_ after treatment (in %)	0	2.1 (1.7/2.0/2.1/2.7)	0	2.0 (1.7/1.9/2.0/2.6)
SD of HbA_1c_ values after treatment (in mmol/mol)	--	--	--	--
SD of HbA_1c_ values after treatment (in %)	0	1.10 (0.20/0.90/1.34/2.93)	0	1.02 (0.10/0.80/1.27/2.75)
Log(SD) of HbA_1c_ values after treatment (in mmol/mol)	--	--	--	--
Log(SD) of HbA_1c_ values after treatment (in %)	0	0.10 (−1.60/−0.11/0.30/1.07)	0	0.02 (−2.30/−0.22/0.24/1.01)

The results from the weighted meta-regression model for assessing the first prerequisite (treatment heterogeneity) are as follows. The key estimate for treatment that measures the difference in log(SD) values between verum and placebo arms is 0.037 (95% CI: 0.004, 0.069). That is, after using the full meta-regression model, we found a slightly larger log(SD) in the verum arms. Estimates for the log(mean) effect were 1.476 (95% CI: 1.128, 1.824), and 0.112 (95% CI: 0.089, 0.147) for the random effects variance.

To put the size of this difference into perspective, we consider the median raw log(SD) across all placebo arms which is 0.10% (see Table 1). This corresponds to a median SD of HbA_1c_ values after placebo treatment of exp(0.10) = 1.105% (12 mmol/mol). An increase of 0.037 on the log-scale would result in an SD of HbA_1c_ values after treatment of exp(0.10 + 0.037) = 1.147% (12.5 mmol/mol). This increase appears rather small if compared with the situation of treatment heterogeneity with two responder groups as given in Fig. 1 where the observed SD is 1.46% (16 mmol/mol).

**Fig. 3:**
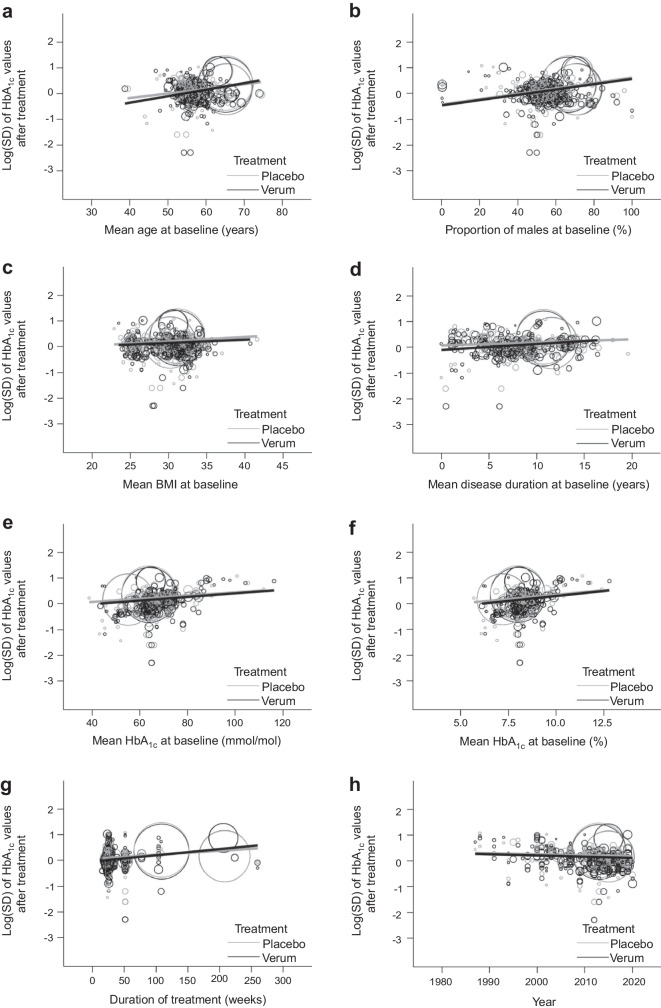
Scatterplots of the log(SD) values of HbA_1c_ values after treatment against continuous predictors. (**a**) Mean age at baseline, (**b**) proportion of male participants at baseline, (**c**) mean BMI at baseline, (**d**) mean disease duration, (**e**, **f**) mean HbA_1c_ at baseline, (**g**) duration of treatment and (**h**) year in the respective treatment arms. Linear weighted fits are given for both treatments, and the two linear regression lines being nonparallel would point to an interaction between the clinical predictor and treatment. Note that the linear fits account for the different weights of trial arms but are not adjusted for mean HbA_1c_ and the correlation within trials

**Fig. 4:**
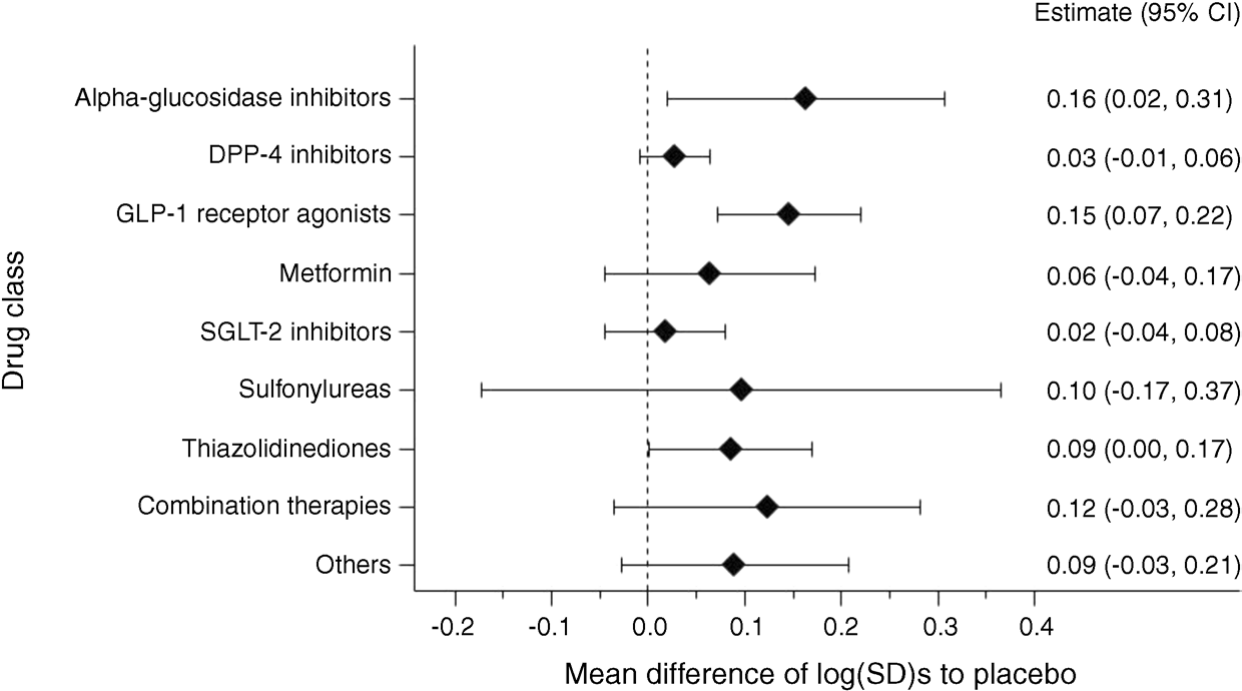
Differences in log(SD) values against placebo for all treatments (drug classes)

The results from the extended weighted meta-regression models for assessing the second prerequisite are given in Fig. 3 and Table 2 (for continuous predictors) and in Fig. 4 for the categorical predictor drug class. Figure 3 shows scatterplots of the log(SD) values against the respective continuous clinical predictor on the x-axis. Again, the regression slopes in Fig. 3 are not adjusted for mean HbA_1c_ and the correlation within trials. We therefore give the fully adjusted slopes of the regression lines and their differences in Table 2. We find no relevant differences between slopes for the clinical predictors, the only exception being the duration of disease where the log(SD) grows faster with increasing disease duration in verum arms.

**Table 2:** Results from assessing the second prerequisite, existence of clinical predictors for the log(SD) of HbA_1c_ values after treatment

Predictor	Number of missing arms for the predictor	Slope of adjusted regression line in:		Slope difference (verum−placebo) (95% CI)
		Verum arms (95% CI)	Placebo arms (95% CI)	
Mean age at baseline (in years)	18	−0.001 (−0.012, 0.010)	0.002 (−0.009, 0.014)	−0.003 (−0.008, 0.003)
Proportion of male participants at baseline (in %)	25	−0.001 (−0.005, 0.002)	0.001 (−0.003, 0.005)	−0.002 (−0.005, 0.0003)
Mean BMI at baseline (in kg/m^2^)	16	0.014 (−0.004, 0.0316)	0.010 (−0.009, 0.028)	0.004 (−0.009, 0.016)
Mean disease duration at baseline (in years)	92	0.007 (−0.009, 0.023)	−0.004 (−0.021, 0.012)	0.011 (0.002, 0.021)
Mean HbA_1c_ at baseline (in mmol/mol)	0	0.006 (−0.001, 0.013)	0.003 (−0.005, 0.010)	0.003 (−0.001, 0.007)
Mean HbA_1c_ at baseline (in %)	0	0.065 (−0.010, 0.140)	0.031 (−0.052, 0.114)	0.034 (−0.008, 0.076)
Duration of treatment (in weeks)	0	0.001 (−0.0005, 0.002)	0.001 (−0.0005, 0.002)	0.00004 (−0.00038, 0.00030)
Year	0	−0.008 (−0.016, 0.0005)	−0.006 (−0.014, 0.003)	−0.002 (−0.008, 0.003)

Each line reports on a separate meta-regression model for each individual predictor. The models are identical to the models for the first prerequisite, however, they were extended by an additional interaction term of the respective predictor with treatment. Given are the slopes of regression lines for the respective predictor in the placebo and the verum arms, as well as their difference, which actually measures the interaction between treatment and predictor

Figure 4 shows differences in outcomes against placebo for all drug classes. Estimates for all drug classes show larger variabilities in verum arms, with the effect most pronounced for alpha-glucosidase inhibitors with an increase of 0.16 (95% CI: 0.02, 0.31) in the log(SD) and for GLP-1 receptor agonists with an increase of 0.15 (95% CI: 0.07, 0.22) in the log(SD) in verum compared with placebo arms.

The analyses for the baseline-corrected log(SD) values as given in the ESM (ESM Results, ESM Tables 1, 2 and ESM Fig. 1–3) essentially replicate the results for the raw log(SD) values. In brief, baseline-corrected log(SD) values were available for 638 trial arms, 405 verum arms with 58,225 participants and 233 placebo arms with 31,784 participants from 229 different trials. In the weighted meta-regression model the estimate for the treatment effect that measured the difference in log(SD) values between the verum and placebo arms was 0.033 (95% CI: −0.002, 0.069), qualitatively identical to the estimate for the raw log(SD) (0.037 (95% CI: 0.004, 0.069)). With respect to the clinical predictors, we also observed larger variability for GLP-1 receptor agonists, with an increase of 0.08 (95% CI: 0.02, 0.13) in the baseline-corrected log(SD) in the verum arms compared with placebo arms.

**Fig. 5:**
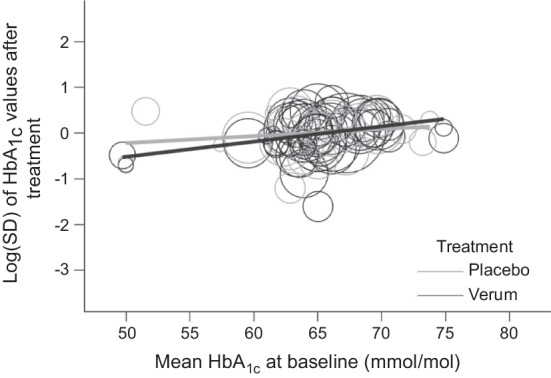
Scatterplot of the log(SD) of HbA_1c_ values after treatment against baseline HbA_1c_ values in the subgroup of studies with GLP-1 receptor agonists. Linear weighted fits are given for both treatments, and the two linear regression lines being nonparallel would point to an interaction between the clinical predictor and treatment. Note that the linear fits account for the different weights of trial arms but are not adjusted for mean HbA_1c_ or the correlation within trials

In view of the large and precisely estimated treatment heterogeneity effects of GLP-1 receptor agonists for both log(SD) outcomes, we repeated the investigation of predictors in the subgroup of trials that assessed GLP-1 receptor agonists. For both log(SD) outcomes there was a clear effect of baseline HbA_1c_ with larger treatment heterogeneity at higher HbA_1c_ values (Fig. 5 and ESM Fig. 4). Thus, there might be potential for use of the precision medicine approach for individuals with poor glycaemic control with a GLP-1 receptor agonist.

## Discussion

This meta-regression of 174 RCTs including 86,940 participants revealed a small increase in the variability of HbA_1c_ values after treatment in the verum arms. Only one out of eight investigated clinical predictors, drug class, was identified as potentially explaining this increase. The potential of the precision medicine approach in the treatment of type 2 diabetes is therefore modest at best, at least with regard to an improvement in glycaemic control as assessed by the HbA_1c_. Nevertheless, the larger variability of response to GLP-1 receptor agonists in individuals with poor glycaemic control, indicated by high baseline HbA_1c_, suggests a potential for precision medicine, which would be of clinical relevance given novel guideline recommendations and the increasing use of this drug class. Of note, GLP-1 receptor agonists exert pleiotropic effects aside from modulating insulin secretion by the incretin effect, such as decreasing appetite, slowing gastric emptying and specific action via receptors in the immune system, heart and kidney [24–26]. These effects and intra-individual variability in its degradation may impact on heterogeneous treatment responses. However, the finding of larger variability of response to GLP-1 receptor agonists needs replication and validation with other clinical outcomes and/or with different study designs.

What is the reason for this unexpected result of rather small treatment heterogeneity that might limit the enthusiasm for precision medicine, at least with regard to the effects of glucose-lowering drugs on glycaemic control? The key point is that we should not view the existing outcome variability in clinical trials or clinical practice as proof that a treated person responds differently to different treatments. What we observe in real life are the effects of treatments *plus* the differences between individuals *plus* the differences within different individuals. However, observing real treatment heterogeneity is more complicated because it entails an interaction effect, i.e. a difference in the effect of treatments in an individual person.

As stated before, heterogeneity of the treatment effect can best be assessed in studies that repeatedly look at the same person receiving at least two different treatments. Even standard crossover trials with each single treatment given only once are of no help here. Instead, we would need repeated-crossover or *N*-of-1 trials that are not yet seen in diabetology. Interestingly, there is a systematic review of *N*-of-1 trials [27] across all clinical disciplines, where the authors indeed found some evidence for the existence of treatment effect heterogeneity. The included trials, however, originated mainly from neurology, rheumatology and psychiatry, and none of them were from diabetology. In addition, Raman et al [27] only reported on the proportion of statistically significant treatment-by-person interactions, but did not aim for an effect measure that would also give an impression of the clinical relevance of these interactions. In view of the treatment heterogeneity observed here for GLP-1 receptor agonists in individuals with poor glycaemic control, one would certainly like to see a trial that compares GLP-1 receptor agonists with a placebo in a repeated-crossover design, preferably also assessing other outcomes such as body weight or diabetes-related complications.

Of course, individual treatment paths with individuals having a treatment more than once can also be collected from observational studies. This would come with the additional advantage of larger external validity, because populations from observational studies are in general more representative than those from clinical trials. However, the challenges of non-randomised data are also well known. Treatment switches would not be randomised but depend on the current glycaemic state of the person, thus potentially confounding the treatment effect and, more importantly, treatment effect heterogeneity.

Our results suggest an intensified search for new, multiomics predictors, e.g. genomics, phenomics or metabolomics for differential treatment response. At least with respect to genomic predictors, such efforts have not yet been very successful [28]. The reason for this is that genetic or genomic factors determining individual response to treatment can only exist if consistent overall individual responses are present. From a statistical point of view, the treatment-by-person interaction, i.e. the individual response to treatment, provides an upper bound to the treatment-by-gene interaction, i.e. the differential response in possible genetic subgroups, because individuals necessarily differ by more than their genes [29]. In addition, type 2 diabetes might be governed mainly by polygenic risks, with individual differences in the contribution of risk alleles and various other factors affecting phenotypes. In this context, one might refer to the recently proposed subclassifications for diabetes, which are based on simple clinical variables and may be useful for future precision diabetology approaches [3].

Beyond the actual empirical analysis, we proposed a general procedure to assess the potential of the precision medicine approach in diabetology. Extending previous analyses in other medical disciplines where only treatment heterogeneity (our first prerequisite) was examined, we argue, following Wilkinson et al [11], that for a complete picture of the precision medicine approach, predictors for treatment heterogeneity should also be investigated. Only if such predictors exist can we identify individuals who would benefit more from a given treatment than from others. Finally, we consider it a strength of the work presented here that we explicitly avoided the ratio-based approach that is regularly used in assessing the potential of precision medicine and instead used the arm-based approach as recommended by Nakagawa et al [23].

We have to acknowledge some limitations of our approach. Foremost, treatment heterogeneity can also exist even though outcome variabilities are similar in the verum and placebo arms; a fictitious example is given in ESM Fig. 5. Therefore, our approach can only yield indirect evidence for the absence of treatment heterogeneity. However, for a situation such as that given in ESM Fig. 5, strong assumptions regarding the correlation between individual placebo and verum responses must be fulfilled [13]. Specifically, those individuals whose HbA_1c_ value would remain unchanged under a placebo treatment would have to show the strongest treatment effect with the verum treatment and vice versa, which is a rather unrealistic assumption. A further limitation is that in trials with more than one verum arm, we did not use the available information on dose; in particular we did not check whether a higher dose of treatment also leads to higher variability; however, this will be the subject of our future work. We restricted the list of investigated predictors to the eight that were available from the three systematic reviews. It is possible that other predictors, e.g. race/ethnicity, treatment adherence, concomitant therapies or lifestyle factors might yield different results. Regarding our clinical outcome of HbA_1c_, it is well known that this is not necessarily a good surrogate for ‘harder’, more clinically- or person-relevant outcomes [22]. Therefore, future work should also investigate other outcomes that are more person-relevant, such as diabetes-related complications or mortality. In addition, GLP-1 receptor agonists and SGLT-2 inhibitors have been shown in RCTs to have beneficial effects on cardiovascular and renal outcomes, which are not necessarily (or only partly) due to their glucose-lowering effects. Thus, further clinical outcomes outside the classical diabetes domain, such as blood pressure, blood lipid levels or renal function, are also candidates for the evaluation of the precision medicine approach. With a view to more formal issues, we did not perform our own trial search, but relied on previous systematic reviews that were chosen because of their recency and the large numbers of included trials. Some trial publications were read by only a single reviewer, and we did not make any attempts to obtain additional information from the authors, e.g. in situations involving missing values.

Finally, a low variability of the clinical outcome in verum arms is not necessarily a disadvantage. Glucose-lowering treatment might have a stabilising quality, eventually shifting HbA_1c_ values across all treated individuals in a small corridor where the harms of too-low as well as too-high values are minimised [10]. Indeed, when we looked at the variances of the mean HbA_1c_ values after treatment in the verum and placebo arms, we found 0.46 (95% CI: 0.41, 0.53) in the verum arms but 0.82 (95% CI: 0.70, 0.97) in the placebo arms, clearly supporting the notion of a stabilising effect of treatment. As such, the potential for precision medicine might be masked by this ‘corridor effect’ when using HbA_1c_ as the clinical outcome. This again emphasises the need for replication of our findings for other clinical outcomes (e.g. body weight or all-cause mortality), which are less prone to this effect because they are not the primary response of diabetes treatment.

Closely related to this, a further reason for this stabilisation of HbA_1c_ values might be the floor effect. Individuals with initial HbA_1c_ values that are already low have a smaller potential for reduction as compared with people starting with high HbA_1c_ values [10]. In addition, the absence of treatment heterogeneity is not necessarily harmful for treated individuals. If the treatments work similarly in all of them, then no one is treated inferiorly.

In conclusion, the overall small differences in HbA_1c_ variability in the verum and placebo arms of RCTs and the absence of predictors for treatment heterogeneity suggest an overall limited potential for the precision medicine approach for glucose-lowering treatment of type 2 diabetes. The promising result we found for GLP-1 receptor agonists in individuals with poor glycaemic control deserves further investigation with other clinical outcomes and/or different study designs. Until then, it is safe to assume that the average treatment effect as observed in standard RCTs is a reasonable expectation for the treated person.

## Supplementary Information

Below is the link to the electronic supplementary material.

Supplementary file1 (PDF 791 KB)

## Data Availability

Two datasets (one for the log[SD] and one for the baseline-corrected log[SD]) to reproduce the analyses from this paper are available on https://zenodo.org/record/7956635.

## Abbreviations

DPP-4: Dipeptidyl peptidase-4
GLP-1: Glucagon-like peptide-1
SGLT-2: Sodium–glucose cotransporter 2

## Authors’ relationships and activities

OK received honoraria for biostatistical education from Berlin-Chemie. SS received a speaker honorarium from Novo Nordisk. MR received personal fees from Allergan, Astra-Zeneca, Bristol-Myers-Squibb, Eli Lilly, Fishawack Group, Gilead Sciences, Intercept Pharma, Inventiva, Novartis, Novo Nordisk, Pfizer, Prosciento, Sanofi US and Target RWE and investigator-initiated research support from Boehringer-Ingelheim, Nutricia/Danone and Sanofi-Aventis. All other authors (MEO, LVB, and AH) declare that there are no relationships or activities that might bias, or be perceived to bias, their work.
