# Supplementary material for: How amenable is type 2 diabetes treatment for precision diabetology? A meta-regression of glycaemic control data from 174 randomised trials
Source: Diabetologia. 2023 Jun 20;66(9):1622–32. doi: 10.1007/s00125-023-05951-2 (PMC10390610; doi:10.1007/s00125-023-05951-2)

Electronic supplementary material

Results for the baseline-corrected log(SD)

From the available number of 377 trials, 144 did not report on the baseline-corrected log(SD), 30 did not report on the sample size and 14 did not report on the mean HbA_{1c} value after treatment. A trial could have no information on more than one of the three items (no log(SD), no sample size, or no mean HbA_{1c} value), so we ended up with a final data set of 638 trial arms (405 verum arms with 58,225 participants and 233 placebo arms with 31,784 participants) from 229 different trials with information on the baseline-corrected log(SD) of HbA_{1c} values after treatment.

The description of trial populations is given in ESM Table 1. At baseline and as a consequence of randomization, trial populations in the placebo and verum arms were similar. Most frequently used treatments were DPP-4 inhibitors (in 103 verum arms), SGLT-2 inhibitors (99), and GLP-1 receptor agonists (93). The median mean HbA_{1c} values (in %) in the trial populations were 65.4/65.6 mmol/mol (8.1/8.2%) *before* treatment, and 63.9/56.5 mmol/mol (8.0/7.3%) in the placebo/verum arms *after* treatment, indicating a clear overall beneficial effect of verum treatments. In terms of the outcome, the median baseline-corrected log(SD) of HbA_{1c} values after treatment was -0.08%/-0.05% in the placebo/verum arms pointing to a minimally larger variability in the verum arms, the full distributions (ESM Fig. 1) of baseline-corrected log(SD) values were very similar in verum and placebo arms.

The results from the weighted meta-regression model for assessing the first prerequisite (treatment heterogeneity) are as follows. The key estimate for treatment that measures the difference in log(SD) values between the verum and placebo arms is 0.033 (95%-CI: -0.002, 0.069), essentially identical to the estimate for the raw

log(SD) as given in the main text which was 0.037 (95%-CI: 0.004, 0.069). That is, after using the full meta-regression model, we found a slightly larger log(SD) in the verum arms. Estimates for the log(Mean) effect were 0.195 (95%-CI: -0.132, 0.521), and 0.086 (95%-CI: 0.070, 0.108) for the random effects variance.

Results from the extended weighted meta-regression model for assessing the second prerequisite are given in ESM Fig. 2 and ESM Table 2 (for continuous predictors) and in ESM Fig. 3 (for the only categorical predictor of drug class). ESM Fig. 2 shows scatterplots of the baseline-corrected log(SD) values against the respective continuous clinical predictor on the x-axis. Linear weighted fits are given for both treatments, and the two linear regression lines being nonparallel would point to an interaction between the clinical predictor and treatment. The regression slopes in ESM Fig. 2 are not adjusted for mean HbA_{1c} and the correlation within trials. We therefore give the fully adjusted slopes of regression lines and their differences in ESM Table 2. We do not find relevant differences between line slopes for the clinical predictors, the only exception being the baseline HbA_{1c} where the baseline-corrected log(SD) grows faster with increasing HbA_{1c} in the verum arms.

In terms of the drug class under study, we point to ESM Fig. 3 where we give the differences in outcomes against placebo for all drug classes. Estimates for most drug classes show larger variabilities in the verum arms. Interestingly and similar to the outcome of raw log(SD) in the main text we see a larger variability for GLP-1 receptor agonists, the increase in the baseline-corrected log(SD) being 0.08 (95%-CI: 0.02, 0.13) in the verum compared to the placebo arms. This confirms the potential of the precision approach for this drug class.

ESM table 1: Description of included trial arms, separated by placebo and verum arms, for the trials reporting on the baseline-corrected log(SD)

	**Placebo (N=233 arms)**		**Verum (N=405 arms)**	
**Variable**	**Number of missing arms**	**Median (Min/Q1/Q3/Max)**	**Number of missing arms**	**Median (Min/Q1/Q3/Max) or Number (%)**
Mean age at baseline (in years)	3	57.1 (39.5/55.0/59.6/74.9)	6	56.5 (38.8/54.6/58.9/74.9)
Proportion of male participants at baseline (in %)	7	54.6 (0.5/49.3/60.9/96.0)	12	54.0 (0.4/47.8/61.5/90.0)
Mean BMI at baseline (in kg/m^2)	7	30.8 (23.8/27.9/32.3/41.6)	14	31.0 (24.0/28.7/32.4/40.7)
Mean known disease duration at baseline (in years)	46	7.6 (0.5/5.7/10.4/19.0)	66	6.9 (0.2/5.2/9.3/19.0)
Mean HbA_{1c} at baseline (in mmol/mol)	0	65.4 (43.4/63.6/69.4/90.2)	0	65.6 (43.5/63.6/69.4/89.1)
Mean HbA_{1c} at baseline (in %)	0	8.1 (6.1/8.0/8.5/10.4)	0	8.2 (6.1/8.0/8.5/10.3)
Year	0	2014 (1995/2009/2017/2020)	0	2013 (1995/2009/2016/2020)
Treatment (drug class)				
Alpha-glucosidase inhibitors	--	--	0	20 (5)
DPP-4 inhibitors	--	--	0	103 (25)
GLP-1 receptor agonists	--	--	0	93 (23)
Metformin	--	--	0	13 (3)
SGLT-2 inhibitors	--	--	0	99 (25)
Sulfonylureas	--	--	0	5 (1)
Thiazolidinediones	--	--	0	41 (10)
Combination therapies	--	--	0	21 (5)
Others	--	--	0	10 (3)

Duration of treatment (in weeks)	0	24<br>(12/24/26/276)	0	24<br>(12/24/26/276)
Number of treated individuals	0	92<br>(10/61/136/4935)	0	111<br>(8/67/156/4932)
Mean HbA_{1c} after treatment (in mmol/mol)	0	63.9<br>(44.3/60.7/68.3/97.8)	0	56.5<br>(35.6/53.7/60.1/88.0)
Mean HbA_{1c} after treatment (in %)	0	8.0<br>(6.2/7.7/8.4/11.1)	0	7.3<br>(5.4/7.1/7.7/10.2)
Log(mean) of HbA_{1c} after treatment (in mmol/mol)	0	4.2<br>(3.8/4.1/4.2/4.6)	0	4.0<br>(3.6/4.0/4.1/4.5)
Log(mean) of HbA_{1c} after treatment (in %)	0	2.1<br>(1.8/2.0/2.1/2.4)	0	2.0<br>(1.7/2.0/2.0/2.3)
SD of baseline-corrected HbA_{1c} values after treatment (in mmol/mol)	--	--	--	--
SD of baseline-corrected HbA_{1c} values after treatment (in %)	0	0.92<br>(0.10/0.78/1.14/2.52)	0	0.95<br>(0.18/0.79/1.16/4.08)
Log(SD) of baseline-corrected HbA_{1c} values after treatment (in mmol/mol)	--	--	--	--
Log(SD) of baseline-corrected HbA_{1c} values after treatment (in %)	0	-0.08<br>(-2.32/-0.25/0.14/0.92)	0	-0.05<br>(-1.70/-0.24/0.15/1.41)

ESM table 2: Results from assessing the second prerequisite, existence of clinical predictors for the baseline-corrected log(SD) of HbA_{1c} values after treatment. Each line reports on a separate meta-regression model for each individual predictor. The models are identical to the models for the first prerequisite; however, they were extended by an additional interaction term of the respective predictor with treatment. Given are the slopes of regression lines for the respective predictor in the placebo and the verum arms, as well as their difference, which actually measures the interaction between treatment and predictor

Predictor	Number of missing arms for the predictor	Slope of adjusted regression line in:		Slope difference (verum-placebo) (95%-CI)
		Verum arms (95%-CI)	Placebo arms (95%-CI)	
Mean age at baseline (in years)	9	-0.010 (-0.018, -0.002)	-0.005 (-0.013, 0.003)	-0.005 (-0.009, 0.001)
Proportion of male participants at baseline (in %)	19	-0.006 (-0.008, -0.003)	-0.004 (-0.007, -0.001)	-0.002 (-0.004, 0.001)
Mean BMI at baseline (in kg/m^2)	21	0.011 (-0.002, 0.024)	0.014 (0.0004, 0.028)	-0.003 (-0.012, 0.005)
Mean disease duration at baseline (in years)	112	0.012 (0.001, 0.024)	0.013 (0.001, 0.025)	-0.001 (-0.008, 0.006)
Mean HbA_{1c} at baseline (in mmol/mol)	0	0.020 (0.014, 0.027)	0.015 (0.008, 0.023)	0.005 (0.001, 0.009)
Mean HbA_{1c} at baseline (in %)	0	0.224 (0.150, 0.297)	0.169 (0.090, 0.247)	0.055 (0.014, 0.096)
Duration of treatment (in weeks)	0	-0.00014 (-0.00168, 0.001403)	-0.00004 (-0.00158, 0.001508)	-0.0001 (-0.0004, 0.0001)
Year	0	-0.007 (-0.014, 0.001)	-0.005 (-0.012, 0.003)	-0.002 (-0.006, 0.002)

ESM figure 1: Boxplots and observed values for the baseline-corrected log(SD) of HbA_{1c} values after treatment, separately for verum and placebo arms. Bottom and top edges of a box display the first (Q1) and third (Q3) quartile, the line inside the box indicates the median value. The red diamond within a box shows the mean value. The whiskers that extend from a box indicate the range of values that are outside of the intra-quartile range (i.e., the difference between Q3 and Q1). Note that these boxplots do not adjust for the mean HbA1c, the sample size or for the correlation within trials

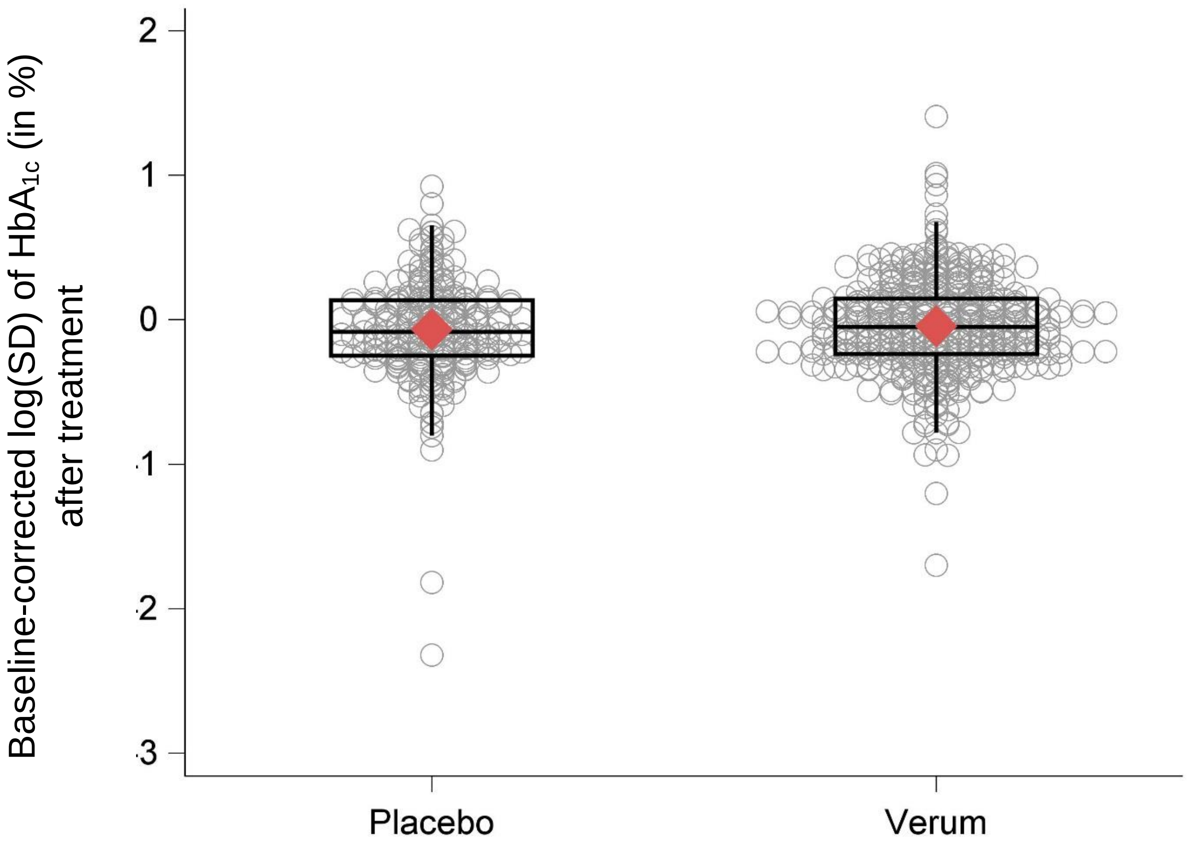

ESM figure 2: Scatterplots of the baseline-corrected log(SD) of HbA_{1c} values after treatment against continuous predictors (**a**) mean age at baseline, (**b**) proportion of male participants at baseline, (**c**) mean BMI at baseline, (**d**) mean disease duration at baseline, (**e**, **f**) mean HbA_{1c} at baseline, (**g**) duration of treatment, and (**h**) year in the respective treatment arms. Linear weighted fits are given for the both treatments, and the two linear regression lines being non-parallel would point to an interaction between the clinical predictor and treatment. Note that the linear fits account for the different weights of trial arms, but are not adjusted for mean HbA_{1c} and the correlation within trials

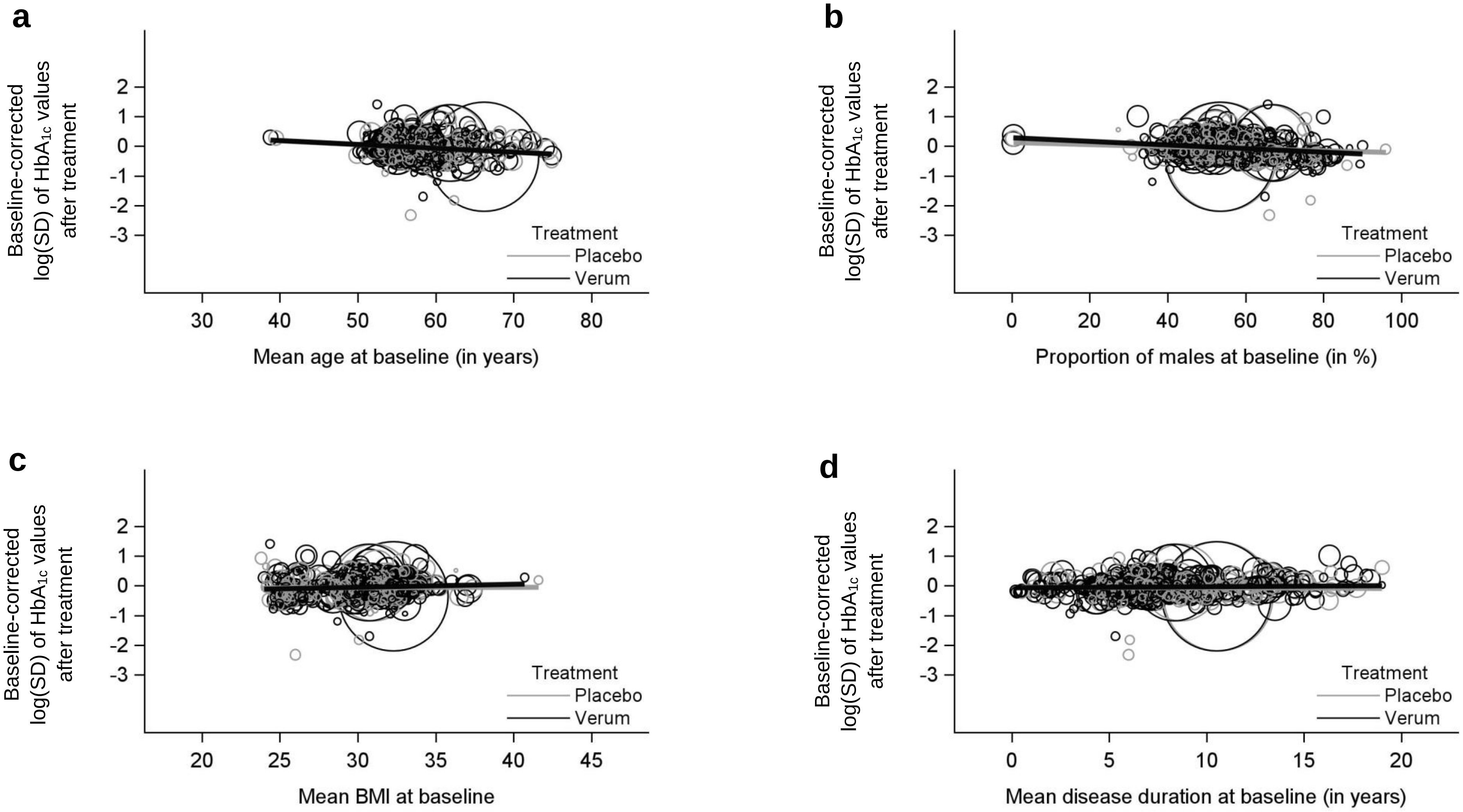

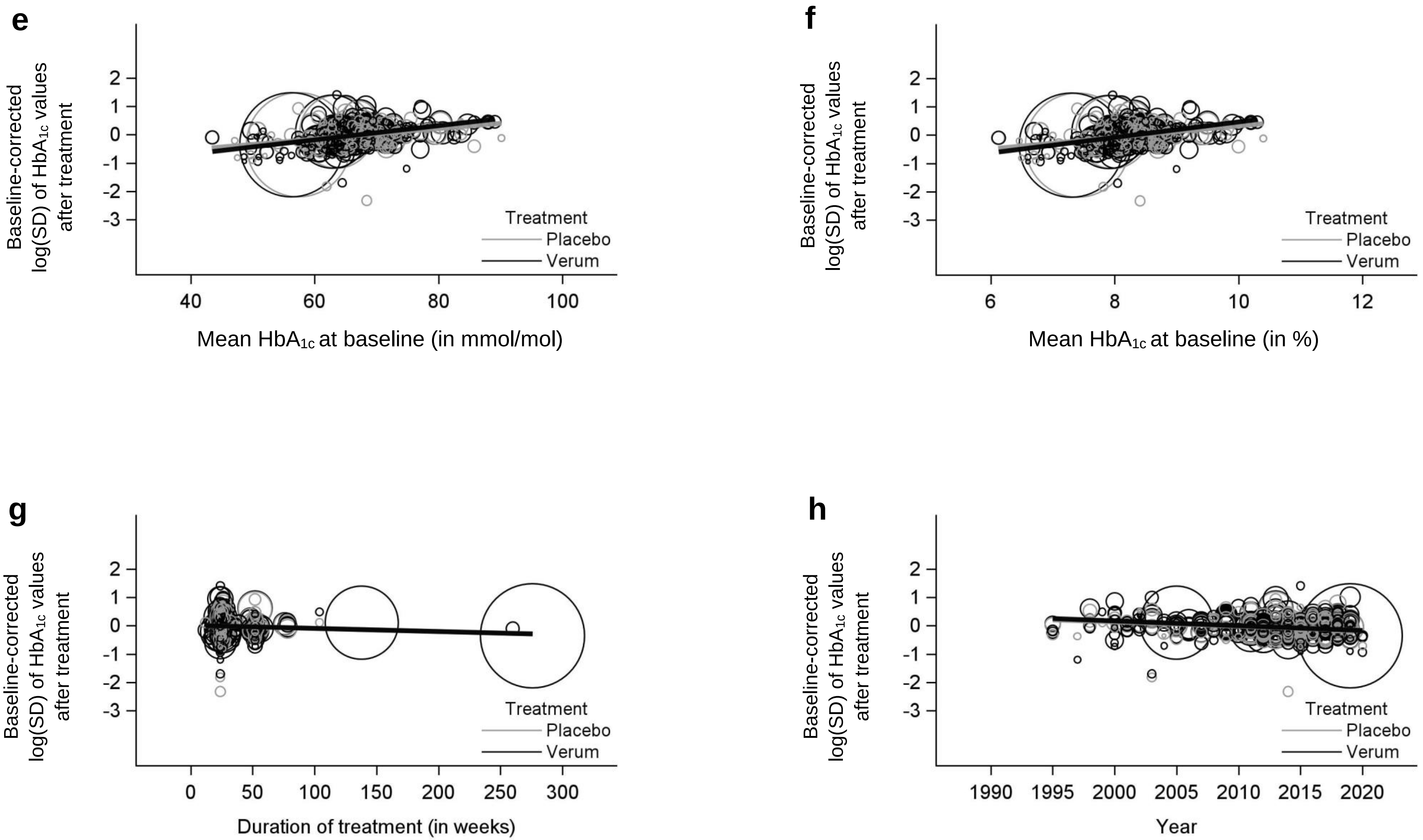
e
Baseline-corrected log(SD) of HbA1c values after treatment
Treatment
Placebo
Verum
Mean HbA1c at baseline (in mmol/mol)
f
Baseline-corrected log(SD) of HbA1c values after treatment
Treatment
Placebo
Verum
Mean HbA1c at baseline (in %)
g
Baseline-corrected log(SD) of HbA1c values after treatment
Treatment
Placebo
Verum
Duration of treatment (in weeks)
h
Baseline-corrected log(SD) of HbA1c values after treatment
Treatment
Placebo
Verum
Year

ESM figure 3: Differences in baseline-corrected log(SD) values against placebo for all treatments (drug classes)

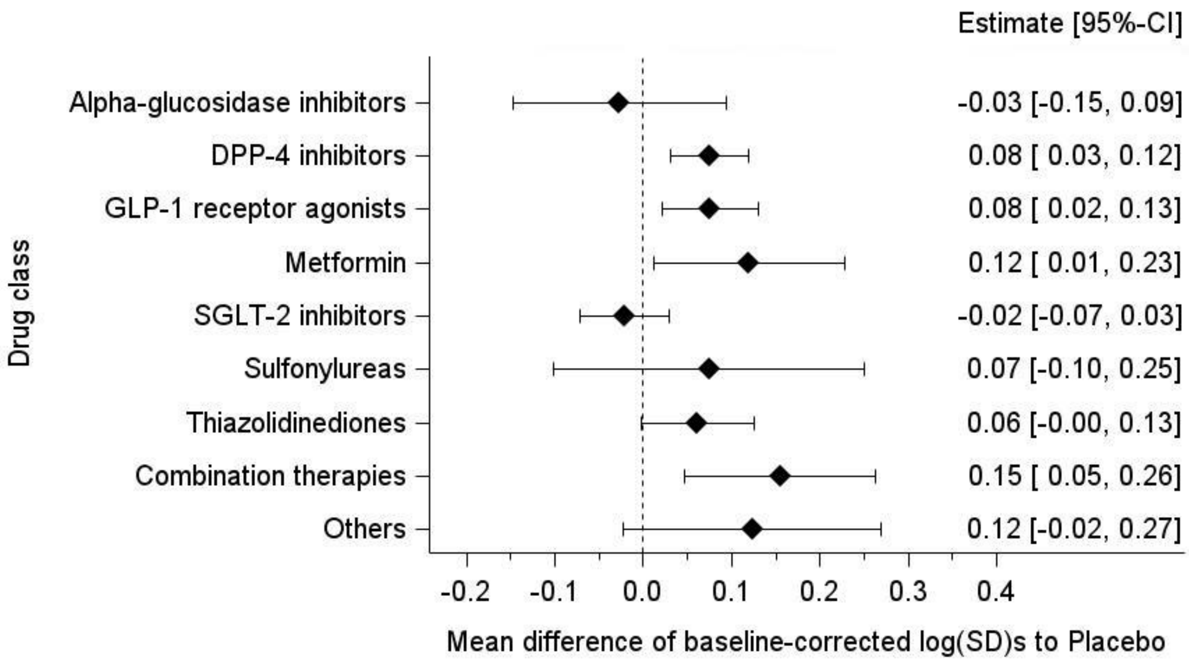

ESM figure 4: Scatterplot of the baseline-corrected log(SD) of HbA_{1c} values after treatment against baseline HbA_{1c} values in the subgroup of studies with GLP-1 receptor agonists. Note that the linear fits account for the different weights of trial arms, but are not adjusted for mean HbA_{1c} and the correlation within trials

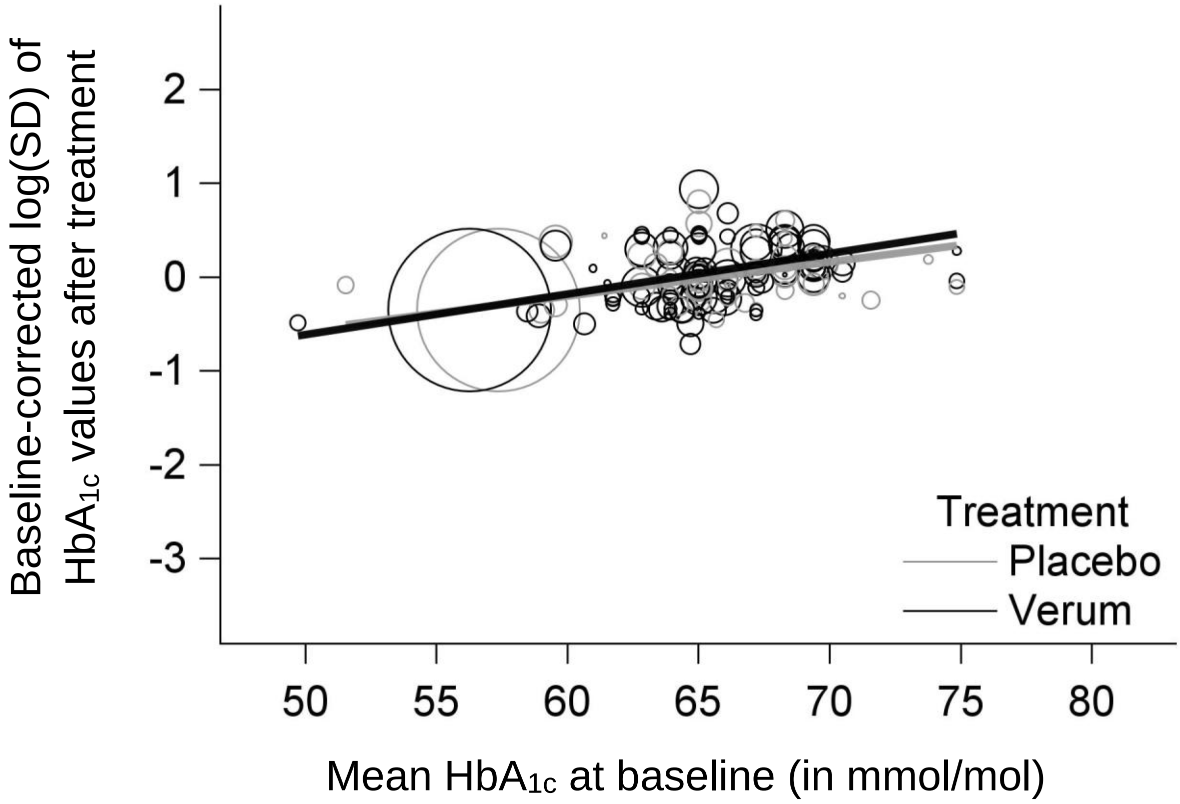

ESM figure 5: Results of a fictious but realistic randomized trial that compares a placebo to a verum treatment. Given are 200 individual HbA_{1c} trajectories from the baseline to the HbA_{1c} value after treatment and (in red) the corresponding standard deviations of HbA_{1c} values at the two different time points and treatment arms.

As a consequence of randomization, the standard deviation of HbA_{1c} values at baseline is identical (=10 mmol/mol (0.92%)) in both arms. There is a treatment effect, the verum treatment leads to a stronger reduction of HbA_{1c} values. There is also treatment heterogeneity between persons. Indeed, there are two groups of responders, a standard responder group (gray lines) and a group of “super-responders” (black lines) in which the HbA_{1c} values are lowered considerably stronger. However, as the variability in the two responder groups is smaller, the overall standard deviation in the verum arm is the same as in the placebo arm (12 mmol/mol (1.10%)).

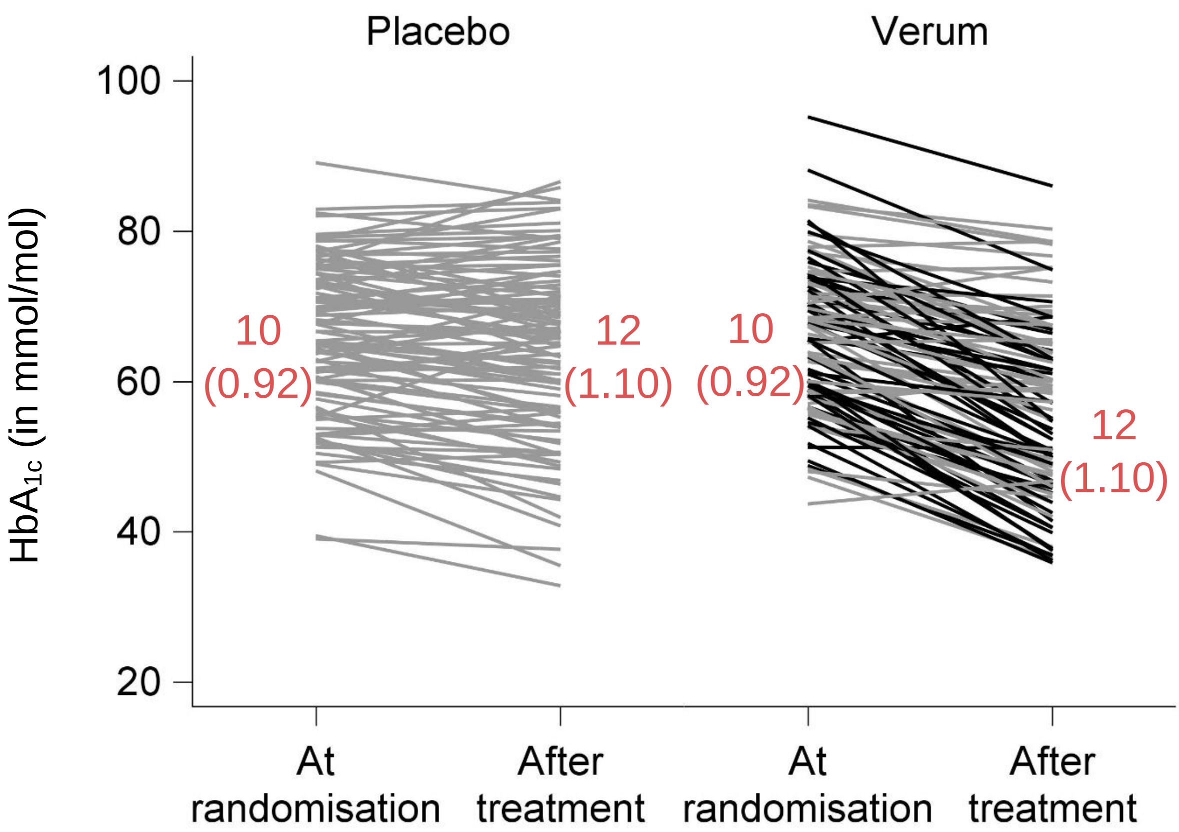

Supplement: Supplementary file 1 — Supplementary file1 (PDF 791 KB) [file 125_2023_5951_MOESM1_ESM.pdf]
